# Management of Cardiopulmonary Complications of Cirrhosis

**DOI:** 10.4061/2011/280569

**Published:** 2011-07-19

**Authors:** Prabha Sawant, C. Vashishtha, M. Nasa

**Affiliations:** Department of Gastroenterology, Lokmanya Tilak Municipal Medical College, Lokmanya Tilak Municipal General Hospital, Sion, Mumbai 400022, India

## Abstract

Advanced portal hypertension accompanying end-stage liver disease results in an altered milieu due to inadequate detoxification of blood from splanchnic circulation by the failing liver. The portosystemic shunts with hepatic dysfunction result in an increased absorption and impaired neutralisation of the gastrointestinal bacteria and endotoxins leads to altered homeostasis with multiorgan dysfunction. The important cardiopulmonary complications are cirrhotic cardiomyopathy, hepatopulmonary syndrome, portopulmonary hypertension, and right-sided hydrothorax.

## 1. Cirrhotic Cardiomyopathy

Cardiovascular abnormalities have been reported by several investigators. Systemic hemodynamic changes occur in cirrhotic patients. There is hyperdynamic circulatory state, decreased arterial blood pressure, decreased peripheral resistance, and increased cardiac output [1]. Because of reduced systemic vascular resistance and increased arterial compliance, left ventricular failure may be latent in cirrhosis. Impaired ventricular function become manifest under strain or treatment with vasoconstrictors. This type of cardiac dysfunction has been termed as cirrhotic cardiomyopathy. 

Three major pathophysiologic abnormalities are observed: cardiac electrophysiological abnormalities, structural and functional ventricular abnormalities, and abnormal ventricular response in presence of pharmacologic, physiologic, or surgical stress. 

Cirrhotic patients have hyperdynamic circulation with decreased peripheral vascular resistance, increased cardiac output and stroke volume, increased organ blood flow, low systemic arterial pressure, and decreased arteriovenous oxygen difference. The level of circulating vasoactive substances which are not inactivated by liver is increased such as vasoactive intestinal peptide, glucagon, tumour necrosis factor-*α*, prostacyclin, nitric oxide, endothelin-1, and endothelin-3 [2]. 

The impaired ventricular response to stress and exercise is due to impaired beta adrenergic signalling pathways, cardiomyocyte dysfunction because of NO overproduction, increased endocannabinoids and carbon mono-oxide, and/or decreased sensitivity to vasoconstrictors (endothelin-1). Increased cell membrane fluidity with beta-receptor dysfunctioning occurs. Endocannabinoids act through CB1 receptors and result in arterial hypotension. CB1 receptor stimulation also enhances the apoptosis of hepatic stellate cells thus producing portal hypertension. Cardiac dysfunction due to local endocannabinoids also occurs [3]. Increased NO production that occurs in cirrhosis decreases vascular responsiveness to vasoconstrictors. NO antagonism can lead to improved responsiveness to vasoconstrictors. There is impaired function of membrane L-type calcium channels. The intracellular storage and release of calcium is not affected. 

There is decreased ventricular systolic response to stress. Cardiac response to exercise is blunted. On stress testing, cirrhotic patients have impaired increase in ejection fraction, chronotropic incompetence, and decreased cardiac index. Impaired cardiac performance occurs in alcoholic and nonalcoholic cirrhotic patients; severity of disturbance depends on degree of hepatic failure [4]. Patients primarily have diastolic dysfunction with left ventricular hypertrophy, left atrial enlargement, isovolumetric relaxation time prolongation, and decreased early to late diastolic flow ratio (E/A ratio). Systolic function of heart is related to heart rate, stroke volume, and cardiac output. During exercise left ventricular dimensions increase because of impaired cardiac systolic function. The right ventricular and pulmonary artery pressure, as well as pulmonary capillary wedge pressure (PCWP) range around upper limit of normal [5]. 

Pathologically, in cirrhotic patients heart weight is increased, dilatation of cardiac chambers, myocardial hypertrophy, and structural changes such as myocardial cell edema, nuclear vacuolation, fibrosis, exudates, and pigmentation occur. The ventricular contractility is regulated by beta adrenergic receptor signalling pathway. The activation of receptor acts through stimulatory G protein (Gsa) with increased cyclic AMP. The cyclic AMP promotes phosphorylation and activation of various cellular proteins by stimulation of protein kinase, with increased intracellular calcium and positive inotropic response [6]. 

The myocardial contractility is regulated by intracellular calcium availability. ATP pumps transfer calcium from cytoplasm into the sarcoplasmic reticulum (SR), the release of calcium from sarcoplasmic reticulum is regulated by calcium channels. Influx of calcium through L-type calcium channels of cell membrane stimulates further release of calcium from the SR. Abnormal functioning of these L-type calcium channels with resultant abnormal release of calcium may explain the abnormality of myocardial contraction in cirrhotic patients [7]. 

Due to altered lipid metabolism in cirrhosis, the cholesterol content of membrane is increased with increased membrane fluidity resulting in desensitization of beta adrenergic receptors. There is decreased Gsa levels in the membrane and increased catecholamines levels in cirrhotic patients. The myocardial performance improves in cirrhotic patients after administration of NO-synthetase inhibitor [8]. 

The increased level of nitric oxide attenuates the activation of L-type of calcium channels. Nitric oxide is produced by NO-synthetase from L-arginine. There is transient bacteremia and increased levels of cytokines and endotoxins, which stimulate the enzyme NO synthetase [9]. Nitric oxide has inhibitory effect on myocardial contractility through increased cGMP. Increased cyclic GMP impair beta adrenergic receptor signalling and calcium release from SR. There is transient bacteremia and endotoxin release in cirrhotic patients with overproduction of cytokines. Carbon monoxide levels are increased in cirrhotic patients. CO induces guanylyl cyclase activity with increased cyclic GMP. Inhibition of heme oxygenase activity can result in improved myocardial contractility [10]. 

There is increased in corrected QT interval. The increased interval correlates with a higher incidence of sudden cardiac death [11]. The pathogenesis of increased QT interval is unclear. The structural changes in cardiomyocyte membrane with increased cholesterol content with resultant membrane fluidity that compromises the calcium and potassium pumps. In cirrhotics increased plasma levels of estrogens has also been implicated for the increased incidence of QT interval prolongation. This interval is increased in 30 to 60% of patients and level of increase relates to degree of hepatic dysfunction. It is also increased in persons with mild portal hypertension thus portosystemic shunting is related to the increase in QTc interval. Increased interval improves with liver transplantation [12]. 

Cirrhotic cardiomyopathy [CCM] remains clinically undetectable but manifests under stressful stimuli. Cirrhotic patients have peripheral vasodilatation thus reduced afterload that prevents development of congestive heart failure. Cardiac dysfunction may become manifest during stressful conditions. Clinical interventions in cirrhotic patients such as during TIPS placement may result in appearance of signs of frank congestive heart failure. 

The patients undergoing liver transplantation may develop pulmonary edema because of cardiac dysfunction together with volume replacement. Thus post transplant patient must have careful fluid replacement because of reduced cardiac reserve. Another complication seen in these post transplant patients is post perfusion syndrome, characterised by decrease of mean arterial pressure of at least 30% for 1 minute within 5 minutes after reperfusion with decrease of heart rate. Likely etiology of which is hyperkalemia, acidosis, and increased tumour necrosis factor-*α*. 

There are no clinical, imaging, or biochemical findings that predict development of CCM so no precise diagnostic criteria has been put forward. As there is no definite diagnostic criteria for CCM treatment guidelines for management have not been clear. Because of the vasodilatation in cirrhotic patients afterload is reduced so cardiac dysfunction remains subtle. In patients with clinically evident heart failure measures include bed rest, supplemental oxygen, and careful use of diuretics. 

The cirrhotic patients have reduced afterload so their tolerability to drugs that decrease preload/afterload is reduced. There is beta receptor signalling defect because of reduced density of receptors in cardiomyocyte membrane. Use of beta agonists such as dobutamine and isoproterenol is less likely to be of benefit. There is increased sympathetic catecholamine stimulation in noncirrhotic heart failure. Thus, the use of beta adrenoreceptor antagonists is preferred over beta agonists in treating noncirrhotic patients with heart failure. Use of aldosterone antagonists such as aldosterone results in ventricular remodelling with reduced left ventricular chamber size and thickness. There is also an improvement in diastolic function with aldosterone antagonists. Orthotopic liver transplantation has been associated with the gradual improvement of the cardiac function over a period of 6 to 12 months. Thus cirrhotic cardiomyopathy represents one of the complication of cirrhosis that can be reversed with the liver transplantation [13].

## 2. Hepatopulmonary Syndrome

This terminology first described in 1977 by Kennedy and Knudson, is defined by classical triad of presence of chronic liver disease or portal hypertension, alteration of arterial oxygenation, defined as widened age corrected alveolar-arterial oxygen gradient with or without arterial hypoxemia and evidence of intrapulmonary vascular dilatations [IPVD] [14]. 

IPVD includes a variety of pulmonary vascular alteration with resultant altered gas exchange due to diffuse peripheral dilatation of pulmonary capillaries. The prevalence of HPS is approximately 10 to 20% in cirrhotic patients evaluated for liver transplant. Patients with cirrhosis should be evaluated for HPS irrespective to the stage of the liver injury. The median survival for HPS without liver transplant is 2 years. The survival is worse if PO_2_ is less than 50 mm Hg, however death is more so due to the complications of liver disease or portal hypertension-related events rather than hypoxemic respiratory failure [15, 16].

In HPS nearly 20% or more of the cardiac output bypasses the functioning alveoli. With exercise this shunt fraction increases. Patients with HPS mostly present with dyspnoea on exertion and subsequently at rest. Most patients will present with the signs and symptoms of liver disease, including gastrointestinal bleeding, esophageal varices, ascites, palmar erythema, and splenomegaly. Digital clubbing, cyanosis, dyspnea, platypnea, and orthodeoxia are the associated other pulmonary signs. Krowka et al. found dyspnea to be the presenting symptom in 18% of patients [17]. Platypnea, defined as dyspnea induced by the upright position and relieved by recumbency [18] and orthodeoxia, defined as arterial deoxygenation accentuated in the upright position and relieved by recumbency [19]. Five percent of patients of cirrhosis have platypnea and orthodeoxia [20, 21]. 

The main cause for severe hypoxemia related to HPS is IPVD. Number of mechanisms have been described in literature. The major mediator of pulmonary vascular abnormality is nitric oxide (NO) a vasodilator molecule guanylyl cyclase in vascular smooth muscle [22, 23]. There is failure of the diseased liver to clear the pulmonary vasodilators. Pulmonary vascular dilatation which results in intrapulmonary shunting is the main determining factor of an impaired gas exchange in HPS and can develop in absence of ascites. There is dilatation of pulmonary precapillary and capillary vessels and there is an alveolar capillary disequilibirium or diffusion-perfusion impairment. The increased diameter of the capillary results in inability of oxygen molecules in adjacent alveoli to diffuse in dilated vessels resulting in impairment of oxygen uptake by RBCs in the central stream of blood vessels [24].

There is also hyperdynamic circulation in the patients with liver disease and there is shortened transit time to the lung vasculature. Bacterial translocation in cirrhosis leads to increased TNF-a, which leads to increased macrophage adherence to pulmonary microvasculature with increased inducible NO-synthase-derived NO production [25]. HPS is not associated with any specific etiology of liver disease and degree of hepatic dysfunction. It should be considered independently of stage of liver disease. HPS has also been diagnosed in noncirrhotic portal hypertension and in liver diseases where portal hypertension is not a feature, such as chronic viral hepatitis without cirrhosis. In patients who do not undergo liver transplantation, the 5-year survival rate is diminished in those who have HPS (20% versus 32–63% without HPS) [26].

## 3. Diagnosis

Several screening algorithms have been proposed. One simple and useful approach is by using pulse oximetry. Oxygen saturation <96% has a sensitivity of 100% and specificity of 88% to detect PaO_2_ < 70 mm Hg and may be used to guide further workup for HPS [27].

In the evaluation of the hypoxemic cirrhotic patient the exclusion of other contributing cardiopulmonary causes such as pulmonary atelectasis, ascites, chronic obstructive pulmonary disease, and hepatic hydrothorax is mandatory. 

Chest radiography shows prominent pulmonary vascular markings in bilateral lower lobes, but finding is not specific. However, a chest X-ray must still be taken to rule out reversible conditions. Similarly, pulmonary function test should be performed to rule out the common intrinsic pulmonary disorders such as chronic obstructive pulmonary disease. 

Contrast echocardiography is the most sensitive test to demonstrate intrapulmonary shunting [28]. It is done using intravenous injections of agitated saline or indocyanine green to produced bubbles of at least 15 microns in diameter. Normally these microbubbles are trapped in the pulmonary vasculature and absorbed. In intracardiac right to left shunts, these microbubbles are seen in the left heart within the first three cardiac cycles [29]. In hepatopulmonary syndrome, because of intrapulmonary shunting, the bubbles are seen in the left heart after the third heart beat, usually between the third and sixth heart beat. Studies have shown that transesophageal echocardiography is more sensitive than transthoracic echocardiography in demonstrating intrapulmonary shunting [30].

There are certain indirect evidences of HPS on echocardiography. A left atrial volume >50 mL is a simple and reliable parameter to detect HPS [31]. Right ventricular diastolic dysfunction is more common in cirrhotic patients with HPS than cirrhotic patients without HPS [32].

There are however a number of limitations of contrast-enhanced echocardiography. It cannot quantify the shunting. It cannot differentiate between intrapulmonary vascular dilatation and direct arteriovenous communication. Although contrast echocardiography is highly sensitive for HPS, it lacks specificity [33]. In patients with concomitant intrinsic lung diseases, contrast echocardiography is a less useful investigation to detect HPS. 

To overcome the disadvantages of low specificity of contrast echocardiography, 99mTechnetium macroaggregated albumin (Tc-99m MAA) lung perfusion scan is used. Albumin macroaggregates with more than 20 *μ*m in diameter, normally are entrapped in the pulmonary vasculature. In patients with intrapulmonary shunts, these albumin macroaggregates escape from the pulmonary vasculature and are taken up by other organs. Normally, less than 5% of isotope reaches brain circulation compared to the lung. In HPS patients, the fraction is more than 6% [34].

In cirrhotic patients with concomitant intrinsic pulmonary disorders, Tc-99m MAA scan can diagnose HPS. However, the major disadvantage of Tc-99m MAA scan is its inability to differentiate intracardiac from intrapulmonary shunting. Pulmonary angiography is another diagnostic modality with potential usefulness [35].

It is however an invasive procedure. Hence, it is reserved for those patients who have a poor response to 100% oxygen, that is, increase in the PaO_2_ to less than 300 mm Hg. Two angiographic patterns have been described. type 1 HPS is characterized by precapillary pulmonary artery dilatation without arteriovenous fistulas. In type 2 HPS there is localized pulmonary arteriovenous fistulous communications. type 1 angiographic findings can vary from normal to diffuse, spider-like, or spongy appearance. type 1 HPS patients with diffuse pulmonary changes have more severe hypoxemia and respond poorly to 100% oxygen. Type 2 HPS is less common. Patients with type 2 HPS do not respond to 100% oxygen. These patients should be considered for embolotherapy although there are case reports of coil embolization in patients with type 1 HPS also. 

Two newer diagnostic modalities for assessing HPS are high-resolution chest computerized tomography (CT) and evaluation of pulmonary blood transit time. The degree of pulmonary microvascular dilation observed on chest CT shows good correlation with the severity of gas exchange abnormalities in patients with HPS. It also helps in quantification of intrapulmonary vasodilatation [36].

Recently, pulmonary transit time of erythrocytes, by using echocardiographic analysis of human serum albumin air microbubble complexes, also correlated with gas exchange abnormalities in a small group of patients with HPS [37]. These two modalities should be tested further in large-scale studies to explore their potential in diagnosis of HPS.

## 4. Therapy

The only established effective therapy for HPS is liver transplantation. There is significant improvement in gas exchange postoperatively in more than 85% of reported patients. However, it may take more than one year for the gas exchange abnormalities to normalise [38].

There is increased mortality after transplantation in patients who have HPS compared with subjects who do not have HPS. Specifically patients with marked hypoxemia (PaO_2_ < 50 mm Hg) and intrapulmonary shunting (shunt fraction > 20%) have increased mortality. Interestingly, unique complications such as pulmonary hypertension, cerebral embolic hemorrhages, and immediate postoperative deoxygenation requiring prolonged mechanical ventilation may contribute to increased postoperative mortality and morbidity [39–42].

Because of the complex relationship between hypoxia secondary to HPS and liver transplantation, MELD exceptions points have been given to patients with HPS and a resting PaO_2_ of <60 mm Hg by the UNOS. Oxygen supplementation, although not studied in the treatment of HPS, is commonly used when PaO_2_ < 60 mm Hg or in conditions with exercise-induced oxygen desaturation. There are anecdotal reports supporting its use demonstrating enhancement of arterial oxygenation, improvement in exercise tolerance, and quality of life. Thus oxygen supplementation is a low-risk treatment option [43].

A number of medical agents have been tried without any robust data showing their benefits. Small uncontrolled studies have shown lack of efficacy using sympathomimetic agents, somatostatin, almitrine, indomethacin, and plasma exchange [44]. An open label trial using garlic also suggests a beneficial effect. In this trial, garlic powder was administered for a minimum of 6 months. Six out of 15 (40%) patients with HPS demonstrated improvements greater than 10 mm Hg in the PaO_2_, and one had even resolution of hypoxemia (PaO_2_: 46–80 mm Hg) over a 1.5-year period [45]. 

Methylene blue infusion, a dye that inhibits the effect of NO on soluble guanylate cyclase, has also shown a transient improvement in oxygenation. Inhaled L-NAME which inhibits nitric oxide production, also transiently has improved oxygenation in one patient (PaO_2_: 52–70 mm Hg), but failed in another group of 10 patients [46]. There is a single case report suggesting that norfloxacin also may be beneficial in improving oxygen saturation in HPS [47].

A few case reports have documented variable improvements in gas exchange using transjugular intrahepatic portosystemic shunts (TIPSs). In a more recent study involving 3 patients with HPS the use of TIPS did not lead to any overall improvement and hence TIPS specifically to treat HPS is not recommended [48].

However, there are reports of success of transcatheter coil embolization of the arteriovenous pulmonary fistulas in type 2 HPS before and after liver transplantation [49]. Embolotherapy may thus be a reasonable first-line option for bridging patients with Type 2 HPS prior to transplantation. Even in patients with type 1 HPS, benefits in reducing morbidity pretransplantation have been described with coil embolization [50].

## 5. Portopulmonary Hypertension

Portopulmonary hypertension [POPH] is defined as pulmonary arterial hypertension, with or without associated liver disease. It was first described by Mantz and Craige in 1951 [51]. The criteria for diagnosis is the presence of portal hypertension, mean pulmonary arterial pressure more than 25 mm Hg at rest with a pulmonary capillary wedge pressure less than 15 mm Hg, associated with the pulmonary vascular resistance greater than 240 dynes·sec·cm^5^.

Most patients of POPH have underlying cirrhosis but it can also develop in noncirrhotic portal hypertension. There is no direct correlation between severity of POPH and etiology or severity of liver disease. POPH is found in 2 to 10% of cirrhotic patients [52–58]. In patients of refractory ascites evaluated for TIPS an unusual high prevalence of POPH of around 16% has been reported. 

Male-to-female ratio is 1.1 : 1. POPH can occur at any age but most commonly presents in fifth decade of life. Diagnosis of portal hypertension precedes the diagnosis of POPH by more than 4 years. The natural history of POPH has not been fully elucidated. Spontaneous resolution of POPH has not been reported. In pretransplant era median survival as low as 6 months was noted. The overall 3-to-5 year survival ranges from 30 to 50% [59, 60].

Death occurs due to complications of liver disease and complications related to POPH in equal proportion of cases. Probability of death due to cardiopulmonary complications is high in those with low cardiac index. An increase in plasma brain natriuretic peptide (BNP) indicates stress on the right ventricle. The differential diagnosis of dyspnea in a patient of liver disease includes intrinsic cardiopulmonary conditions such as chronic obstructive pulmonary disease, pneumonia, pulmonary embolism, congestive heart failure, valvular heart disease, and conditions related to underlying liver disease and portal hypertension such as ascites, hepatic hydrothorax, and muscle wasting [61, 62].

Arterial blood gas analysis shows hypocapnia, an increased alveolar-arterial oxygen gradient and mild hypoxemia. The X-ray chest may show cardiomegaly and prominent main pulmonary artery. 

POPH is graded according to the degree of elevation of mean pulmonary arterial pressure. Mild POPH (mPAP = 25–35 mm Hg) is not associated with increased operative risk for liver transplantation and may not require medical therapy. Moderate POPH (mPAP = 35–50 mm Hg) has increased operative risk for liver transplantation and requires medical therapy. Severe POPH (mPAP > 50 mm Hg) has high operative mortality and is managed with medical therapy [52]. Histologically POPH has medial proliferation and hypertrophy, plexiform arteriopathy, and in situ vascular thrombosis of pulmonary vasculature. Cirrhosis is associated with hyperdynamic circulation with increased shearing stress on pulmonary vasculature with resultant progressive pulmonary vascular remodelling and thrombosis. There is an imbalance between vasodilators and vasoconstrictors. The associated bowel wall congestion due to splanchnic vasodilatation leads to the release of endotoxins such as endothelin-1 and thromboxane [58, 63, 64].

The most common symptom is exertional dyspnoea, other symptoms like chest discomfort, fatigue, syncope, and light headedness may also occur. The signs include elevated jugular venous pressure, loud second pulmonic heart sound, murmur of tricuspid regurgitation, and lower extremity edema. Peripheral edema out of proportion to degree of ascites in a cirrhotic patient, right ventricular dysfunction secondary to pulmonary hypertension should be considered [65, 66].

Transthoracic echocardiography is the recommended screening test. It evaluates right heart function and estimates the right ventricular systolic pressure. Echocardiography excludes valvular heart disease and other causes of elevated mPAP. Echocardiography may reveal changes due to raised resistance to pulmonary flow such as pulmonary valvular insufficiency, right atrial dilatation, right ventricular hypertrophy and dilatation, interventricular septal thickening, and paradoxical movement of septum. The correlation of right ventricular pressure measured during echocardiography and that from right heart catheterization is not good. As echocardiography cannot estimate pulmonary vascular resistance, approximately 30–40% of patients with estimated right ventricular systolic pressure threshold can have normal pulmonary vascular resistance during right heart catheterization and will not be diagnosed as POPH [67].

Another parameter measured during echocardiography evaluation is pulmonary acceleration time. The value of pulmonary acceleration time greater than 100 m sec indicates POPH. Echocardiography apart from the screening is also helpful in followup of patients with POPH. 

Right heart catheterization helps in estimation of mPAP, pulmonary capillary wedge pressure, and cardiac output; calculation of pulmonary and systemic vascular resistance. Patients of liver disease have hyperdynamic circulation and volume overload. Those with pulmonary capillary wedge pressure greater than 15 mm Hg, the diagnosis of POPH may be missed. The use of transpulmonary pressure gradient helps in identifying patients with obstruction to flow, independent of pulmonary capillary wedge pressure. It is calculated by subtracting pulmonary wedge pressure from mean pulmonary arterial pressure [68]. Right ventricular systolic pressure greater than 40 mm Hg or presence of right ventricular abnormalities support further evaluation for POPH. In all patients with echocardiographic abnormalities suggestive of POPH, pulmonary artery catheterization is performed to establish diagnosis and assess severity of POPH. 

Vasodilator testing using either inhaled nitric oxide or intravenous epoprostenol during right heart catheterization may be done. If the diagnosis of POPH is made, a decrease in mPAP and PVR more than 20% from baseline without decrease in cardiac output indicates reversible vasoconstriction. Studies on long-term pharmacologic management of POPH are lacking.

## 6. Medical Therapy

Supplemental oxygen is commenced if hypoxemia is present. Diuretics are used for volume overload. If patients are on B blockers, drugs are to be withdrawn. Treat the varices with band ligation. Endothelin receptor antagonist, Bosentan is dual ETA and ETB receptor antagonist given orally. It is started at the dose of 62.5 mg twice daily and then the dose can be increased to 125 to 250 mg twice daily [69]. Dose-dependant increase of liver enzymes is seen because of inhibition of bile salt export protein. Treatment with low-to-medium dose bosentan improves exercise capacity and pulmonary hemodynamics. Phosphodiesterase inhibitor, sildenafil, inhibits the enzyme phosphodiesterase-5. inhibition of degradation of NO promotes vasodilatation, but may exacerbate portal hypertension and hyperdynamic circulation [70].

Prostacyclin analogue, esoprostenol, is a potent systemic and pulmonary vasodilator with antiplatelet aggregating properties. It has a half-life of 3 to 5 minutes so requires long-term continuous intravenous infusion [71]. It improves pulmonary hemodynamic status. In some transplant centres this may improve patient's status for listing for liver transplantation. Common side effects include headache, flushing, diarrhoea, and hypotension [72]. More stable analogues such as iloprost and treprostinil are being investigated [73, 74].

Denovo POPH, transition from HPS to POPH and recurrences of POPH in cases of graft failure have been noted after LT. All candidates for liver transplantation should undergo screening for portopulmonary hypertension by echocardiography. If the echocardiography shows elevated pulmonary arterial pressures, right heart catheterization is performed to confirm the diagnosis. The ideal medical regimen remains to be determined. Although drug treatment may lower pulmonary artery pressures in selected patients so that liver transplantation can be safely done, morbidity and mortality rates are higher in patients with moderate-to-severe portopulmonary hypertension [75]. Moderate-to-severe POPH with mPAP > 50 mm Hg is contraindication to LT. Liver transplantation is not the treatment of choice for portopulmonary hypertension.

## 7. Hepatic Hydrothorax

Hepatic hydrothorax is defined as the presence of pleural fluid (usually greater than 500 cc) in a patient with cirrhosis after ruling out primary cardiac or pulmonary disease. This occurs in approximately 6–10% of patients with advanced cirrhosis [76]. It is more commonly associated with alcohol-induced liver disease and with the concomitant presence of ascites. Regarding the side of involvement in hepatic hydrothorax, 85% have been right sided, 13% left sided, and 2% bilateral [77].

## 8. Pathogenesis

Several mechanisms have been postulated for the development of hepatic hydrothorax. The direct passage of peritoneal fluid via diaphragmatic defects appears to be the most acceptable explanation. Lieberman et al. demonstrated the defects by introducing CO_2_ into the peritoneal cavity of patients with hepatic hydrothorax [78].

A pneumothorax indicative of a diaphragmatic defect was seen in these patients on chest radiographs, taken within 48 hours. Intraperitoneal injection of methylene blue can be used intraoperatively to localize the defect(s). 

Several scintigraphic studies using intraperitoneal instillation of 99mTc-human serum albumin or 99mTc-sulphor-colloid have demonstrated radioactivity in the pleural cavity minutes to hours after administration [79, 80]. 

The movement of radioisotope is unidirectional towards the pleural cavity due to negative intrathoracic pressure compared to increased intra-abdominal pressure. Microscopic examinations of these defects have showed gaps in the collagen bundles in the tendinous portion of the diaphragm. In patients of ascites, there is increase in the intra-abdominal pressure. This tends to stretch the diaphragm; thereby, creating or enlarging these microscopic defects. The increase in abdominal pressure results in herniation of peritoneum through these gaps in the pleural cavity. This leads to the formation of pleuroperitoneal blebs. These blebs tend to rupture, creating free communication between the peritoneal and pleural cavities. For certain unknown reasons the left hemidiaphragm is more muscular and relatively resistant to blebs formation.

## 9. Clinical Features

It may simply be an incidental finding on a chest radiograph performed for unrelated reasons in a patient of cirrhosis. However, a small subset of cirrhotic patients may present primarily with pulmonary complaints related to hydrothorax such as dyspnea, nonproductive cough, pleuritic chest pain, or fatigue related to hypoxemia [81]. With large pleural effusions severe dyspnea and potential respiratory compromise can occur. 

There are several causes of pleural effusion in general and patients of cirrhosis can have any of those. In a study on patients with end-stage liver disease patients with pleural effusions 30% of patients upon thoracentesis yielded a diagnosis other than hepatic hydrothorax including spontaneous bacterial empyema (SBEM), tuberculosis, adenocarcinoma, parapneumonic empyema, and undiagnosed exudates [82]. Hence both thoracentesis and paracentesis should be performed to ascertain that both fluids are similar in character [83].

The composition of pleural fluid from hepatic hydrothorax, is similar to that of ascitic fluid. However, ascitic and pleural fluid analysis may not be completely identical, perhaps due to the greater efficacy of water absorption by the pleural surface. The cell count is usually low, and the total protein, albumin, cholesterol, and total lipid levels may be marginally higher in the pleural fluid compared to ascitic fluid [84]. However, the serum-to-pleural fluid albumin gradient is usually greater than 1.1 g/dL although, this has not been studied extensively. 

SBEM is the infection of a preexisting pleural effusion (hydrothorax) in a patient with cirrhosis. Its incidence is around 15% (similar to the incidence reported for spontaneous bacterial peritonitis; SBP) in cirrhotic patients with ascites [85]. 

Its pathogenesis is also similar to that of SBP. The diagnosis of SBEM is made if the pleural fluid (PF) cultures are positive and a polymorphonuclear (PMN) count is >250 cells/*μ*L. If culture is negative (and compatible clinical course) the diagnosis is made with a pleural fluid PMN count >500 cells/*μ*L and by excluding a parapneumonic infection [86].

The microorganisms responsible for SBEM appear similar to that of SBP. Patient can present with local symptoms such as dyspnea or pleuritic chest pain, or with systemic symptoms such as fever, shock, or encephalopathy. Up to 40% of SBEM cases may not be associated with SBP. The treatment of SBEM is similar to that of SBP [87]. Despite treatment, mortality remains high at approximately 20%. Albumin therapy at 1.5 g/kg on day 1 and 1.0 g/kg on day 3 in the setting of SBEM may be considered although albumin infusion has not been specifically studied in the setting of hepatic hydrothorax and SBEM.

## 10. Diagnosis

This entity is usually suspected in a patient of cirrhosis if patient presents with pulmonary symptoms or features suggestive of pleural effusion on examination or on routine chest radiographs. A computerized tomographic (CT) scan of the chest should be obtained to exclude any mediastinal, pulmonary, or pleural pathology. 

Moreover, detailed information of the diaphragm may be obtained with a CT scan or a magnetic resonance imaging, permitting recognition of the small diaphragmatic defects [88].

Thoracoscopy may also reveal the defects, but this procedure is invasive and carries significant morbidity in patients with advanced liver disease and therefore is rarely performed. Echocardiography may be indicated if there is a suspicion of pericardial or a cardiac pathology. In difficult cases, specifically when ascites is not detected or the hydrothorax is present on the left side; an intraperitoneal injection of [99Tcm] sulphur colloid or [99Tcm]-human serum albumin may be helpful. Migration of the radioisotopes from the peritoneal cavity into the pleural space establishes a communication between both spaces and confirms that the ascites is the source of the effusion [89, 90]. Conversely, failure of the marker to show up in the pleural space indicates an alternate diagnosis for the pleural effusion. This test has been considered the gold standard for identification of hepatic hydrothorax due to its very high specificity (up to 100%). However, its sensitivity remains modest (approximately 71%). Fortunately, the sensitivity of the test can be greatly improved (up to 100%) by performing a thoracentesis prior to administration of radioisotopes in order to reduce pleural pressure [91].

## 11. Treatment

The first and most important aspect in the management of all patients with cirrhosis and ascites or hepatic hydrothorax is evaluation for candidacy for liver transplantation. Patients of hepatic hydrothorax can be managed by dietary, pharmacologic, and radiological interventions. In selective patients with refractory hydrothorax, surgical approaches aimed at repairing the diaphragmatic defects responsible for pleural fluid accumulation can be considered.

## 12. Diet and Pharmacological Management

It is similar to the therapy of ascites. Achieving a negative sodium balance is the primary goal of dietary and pharmacologic management. Dietary restriction of sodium intake to 2 g/d (88 mEq/d) is the simplest manner by which achieve a negative sodium balance can be achieved [92].

However, most patients with ascites, and almost all patients with hepatic hydrothorax require diuretics (spironolactone and/or furosemide) along with salt restriction. These diuretics are maintained at a ratio of 10 : 4 (spironolactone 100 mg: furosemide 40 mg) to avoid dyselectrolytemia and dosages are increased as needed to attain a goal of producing renal excretion of at least 120 mEq of sodium per day [93].

Patients not responding despite fluid and sodium restriction and use of maximal tolerable doses of diuretics are considered to have refractory hydrothorax. Approximately 10% of patients either do not respond to diuretic therapy or develop diuretic-induced complications that prevent the use of high doses of these drugs. These patients should be considered for orthotopic liver transplantation. Other agents such as terlipressin, octreotide, and midodrine have been used in small studies with moderate benefit [94–96]. These agents will reduce splanchnic blood flow and hence decrease peritoneal and pleural fluid accumulation. However, presently there is not enough evidence to recommend routine use of these agents.

## 13. Thoracocentesis

It is a simple and relatively safe procedure which can be performed in patients with dyspnea for immediate relief of symptoms. In patients with dyspnea and both hepatic hydrothorax and massive ascites, it is recommended to drain the ascites prior to performing a thoracocentesis. It is recommended that no more than 2 liters of fluid should be removed during the first therapeutic thoracocentesis, in order to minimize the risk of unilateral pulmonary edema and/or hypotension [97]. The utility of concomitant albumin infusion has not been established. However, given the relatively small volume of fluid removed at thoracentesis, intravenous albumin to avoid circulatory dysfunction unlike its routine use with large volume paracentesis seems unnecessary. The major risk of thoracocentesis is development of pneumothorax. Usually diagnostic thoracocentesis carries a low risk (1%) of pneumothorax, compared to therapeutic thoracocentesis where the incidence is nearly 9% [82]. 

However, when thoracocentesis is required too frequently (<every 2-3 weeks) in patients on maximal sodium restriction and optimal diuretics, alternative treatment options must be considered.

## 14. Radiologic Interventions: Transjugular Intrahepatic Portosystemic Shunts (TIPS)

It is a nonselective side-to-side portosystemic shunt which decreases the sinusoidal hypertension that leads to ascites formation—an essential step for pleural fluid accumulation. In a study by Gordon et al. 24 Child class B and C cirrhosis patients following TIPS placement were evaluated. Fourteen out of twenty-four (58.3%) had complete resolution of symptoms following TIPS and did not require further thoracocentesis. Another 5 (20.8%) required fewer number of thoracentesis. But, despite this superior efficacy, 6 patients (25%) died of either postprocedure complications (1/6) or liver failure (5/6), and 9 (37.5%) developed transient hepatic encephalopathy [98]. Other groups have also showed symptomatic improvement in many patients, but with associated complications and did not improve the overall prognosis. A recent study has shown that severity of liver dysfunction is directly related to nonresponsiveness and higher one-year mortality after TIPS placement for refractory HH [99].

Thus, it should be considered in selected patients who reaccumulate their effusions rapidly (despite medical treatment) with a Child-Pugh score of less than 10, are younger than 60 and do not have hepatic encephalopathy or severe pulmonary hypertension.

## 15. Surgery

Pleurodesis: Falchuk et al. first described the use of tetracycline-induced pleurodesis in 2 patients with recurrent hepatic hydrothorax. In that study, one patient remained free of effusion at 6-month followup while the other died of variceal hemorrhage 3 weeks after the pleurodesis [100]. Chemical pleurodesis is not always successful and has a modest risk of complications such as fever, chest pain, empyema, incomplete reexpansion, pneumonia, and wound infection. Hence pleurodesis by itself is rarely performed and is reserved for patients in whom no other options exist. 

Interestingly, the use of continuous positive airway pressure (CPAP) appears effective in keeping the pleural cavity dry after chemical pleurodesis. The underlying mechanism postulated is that CPAP will decrease the negative pleural pressure and thus prevent the shift of fluid form the peritoneal to the pleural space [101]. Further studies are needed before this can be routinely recommended.

**Chest Tube Placement**

It leads to massive fluid shifts, protein, and electrolyte depletion. Hence, chest tube insertion is considered a relative contraindication for the treatment of hepatic hydrothorax [102].

**Repair of Diaphragmatic Defects**

Thoracoscopy to repair diaphragmatic defects with/without sclerosing the pleural membranes is a good alternative in patients with refractory hepatic hydrothorax who are not candidates for TIPS. Thoracoscopy appears to be more likely to be effective if diaphragmatic defects can be identified. In a study by Mouroux et al. using video-assisted thoracoscopy (VATS) to close large defects using sutures and biologic glue in combination with talc pleurodesis in 8 patients. None of the patients (6/8) with repaired defects developed recurrent hydrothorax despite the recurrence of ascites [103]. Two other studies (15 and 41 patients each) showed almost 75% success rate with VATS-assisted talc pleurodesis without resorting to diaphragmatic repairs. Thus it may be considered a palliative alternative not only to patients requiring frequent thoracentesis, but also an alternative to TIPS [104, 105].

**Peritoneovenous Shunts**

A peritoneovenous shunt (Le Veen shunt) to divert ascitic fluid has been used in the past in refractory cases. However, the shunt is rendered ineffective over time as the intrathoracic pressure is lower than the central venous pressure resulting in fluid flow towards the pleural space [106]. Because of this reason and frequent complications associated with LeVeen shunt (infection, coagulopathy, and bleeding in compromised host), this procedure has become almost obsolete.

**Liver Transplantation**

It is the only option available when all other therapies fail and is curative for most patients with this complication. The short-term and long-term prognosis in patients undergoing liver transplantation for refractory hepatic hydrothorax appears similar to other groups [107].
